# Expression of neuropilin-1 is linked to glioma associated microglia and macrophages and correlates with unfavorable prognosis in high grade gliomas

**DOI:** 10.18632/oncotarget.26273

**Published:** 2018-11-02

**Authors:** Michael D. Caponegro, Richard A. Moffitt, Stella E. Tsirka

**Affiliations:** ^1^ Program in Molecular and Cellular Pharmacology, Department of Pharmacological Sciences, Stony Brook University, Stony Brook, NY, USA; ^2^ Department of Biomedical Informatics, Stony Brook University, Stony Brook, NY, USA; ^3^ Department of Pathology, Stony Brook University, Stony Brook, NY, USA

**Keywords:** neuropilin, GAM, TCGA, glioma, glioblastoma

## Abstract

High grade gliomas, including glioblastoma (GB), are devastating malignancies with very poor prognosis. Over the course of the last decade, there has been a failure to develop new treatments for GB. Reasons for this failure include the lack of validation of novel molecular targets, which are often characterized in animal models and directly transposed to human trials. Here we build on our previous findings, which describe how the multi-functional co-receptor Neuropilin-1 (NRP1) signals through glioma associated microglia/macrophages (GAMS) to promote murine glioma, and investigate NRP1 expression in human glioma. Clinical and gene expression data were obtained via The Cancer Genome Atlas (TCGA), and analyzed using R statistical software. Additionally, CIBERSORT *in silico* deconvolution was used to determine fractions of immune cell sub-populations within the gene expression datasets. We find that NRP1 expression is correlated with poor prognosis, glioma grade, and associates with the mesenchymal GB subtype. In human GB, NRP1 expression is highly correlated with markers of monocytes/macrophages, as well as genes that contribute to the pro-tumorigenic phenotype of these cells.

## INTRODUCTION

Gliomas account for 60% of all primary and other CNS tumor diagnoses, and make up ~80% of all malignant brain tumors [1]. The World Health Organization (WHO) classifies gliomas by histology and molecular subtype, and on a grading scale of I, II, III, IV. Low Grade Gliomas (LGG) typically range from grades I–III, while High Grade Gliomas (HGG) are categorized as grades III–IV. Glioblastoma (GB) is a grade IV glioma subtype which often spontaneously arises in the CNS, but can also progress from LGG [2]. GB represents a staggering 50% of all malignant brain tumors, making it the most common adult CNS malignancy [1, 3]. With the current standard of care, the median survival of patients diagnosed with GB is dismal, approximately 14–15 months [4]. The high failure rate of phase III interventional clinical trials for GB highlights concerns with the predictive power of animal models, as well as the rapid development of resistance supported by the evolving neoplasm. To aid in the development of future therapeutics for GB, novel molecular targets need to be fully validated and established.

Neuropilin-1 (NRP1) is a multi-functional co-receptor present in most tissues. It is expressed by a diverse range of cell types, such as neurons, glia, endothelial and immune cells. NRP1 was originally reported to associate with the cell surface receptor Plexin A1 and facilitate ligation with SEMA3A in neurons, which drives axonal pathfinding [5]. NRP1 has also been identified to complex with transforming growth factor β receptor I/II (TGFβRI/II), vascular endothelial growth factor receptors (VEGFR), and hepatocyte growth factor receptor (cMET) [6–8]. NRP1 is variably expressed in malignancies, where it functions to promote VEGF-dependent angiogenesis and endothelial cell migration [9].

The complex tumor microenvironment (TME) of the brain is made up of non-cancerous stromal cells such as endothelial cells, cells of the immune system, astrocytes and microglia - all of which contribute to the maintenance and growth of malignant gliomas [10]. Under homeostatic conditions, resident microglia of the brain and systemic monocytes and macrophages from the periphery survey the body for pathogenic threats, and act as effector cells of the innate immune system. However, in GB, glioma associated microglia and macrophages (GAMs) traffic to cancerous lesions, where they become subverted by tumor cells and are largely responsible for orchestrating tumor progression by secreting factors promoting chemoattraction, immune suppression, neoangiogenesis, and tumor cell survival (reviewed in [11, 12]). Pro-tumorigenic GAMs are enriched in HGG and found in all GB molecular subtypes [13–15]. GAMs are now recognized as an integral part of glioma progression, and as such, pre-clinical therapies that modulate these cell populations aim to harness their properties to slow or reverse tumor growth [16–18].

NRP1 signaling in monocytic cells, such as GAMS, is not well characterized. In models of lung, breast and pancreatic cancers, macrophage-specific deletion of NRP1 shows profound effects on disease progression [19]. Lack of NRP1 signaling in LysM^+^ cells caused a shift in localization away from hypoxic areas, reduced neoangiogenic function, and reversed the immunosuppressed TME [19]. Our lab has previously shown that GAM-specific ablation of NRP1, or global pharmacological inhibition of this co-receptor, slows tumor progression in a mouse model of GB, in a similar fashion, by inhibiting neoangiogenesis and reducing immunosuppressive signaling [20]. Further, we reported that either population of microglia or peripheral macrophages lacking NRP1 were sufficient to inhibit disease progression in this model [21]. We have also shown qualitatively that NRP1 co-localizes with the pan-monocyte marker Iba1 across all glioma grades in archived human biopsies [20]. Thus, monocytic NRP1 may be a potentially exploitable therapeutic target for human GB.

The association between NRP1 expression and patient prognosis is not well studied in glioma. In 2004, Osada *et al.* reported that patients overexpressing NRP1 have poor prognosis [22], however, the study was limited by small cohort size and mixed glioma diagnoses. Further, relative expression levels were not investigated.

To establish NRP1 as a therapeutic target in GB, this correlation must be corroborated. Whether NRP1 expression by GAMs drives their pro-tumorigenic phenotype and/or glioma disease progression in humans is unknown. We describe here our analysis of two questions: 1) Is NRP1 expression correlated with glioma prognosis and 2) Can NRP1 expression be linked to pro-tumorigenic GAMs of the TME.

## RESULTS

### NRP1 expression correlates with poor prognosis and clinicopathological features in glioma

**Figure 1:**
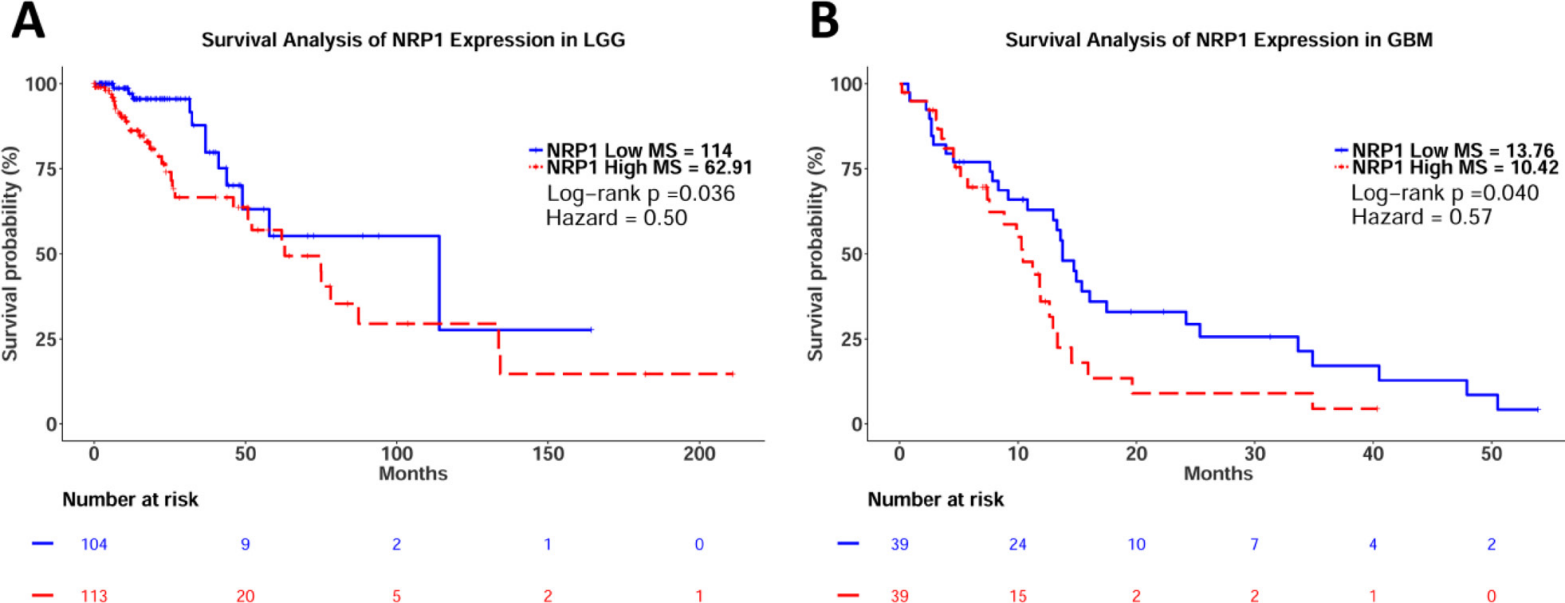
Kaplan–Meier survival analysis of relative NRP1 expression in LGG and GB (**A**) LGG Cohort. NRP1 Low median survival = 114 months, *n* = 105, events = 12. NRP1 High median survival = 62.91 months, *n* = 114, events = 32. Log-rank *p* val = 0.036, HZ = 0.50 (0.25–0.97). (**B**) GBM Cohort. NRP1 Low median survival = 13.76 months, *n* = 39, events = 31. NRP1 High median survival = 10.42, *n* = 39, events = 27. Log-rank *p* val = 0.040, HZ = 0.57 (0.33–0.98).

Based on our animal data [20, 21, 23], we sought to make meaningful extrapolations about NRP1 expression in human glioma. TCGA database enabled the mining of publicly available data before embarking on large scale human projects. TCGA is a robust tool used in many bioinformatical analyses to investigate connections between gene expression, mutations, demographics and clinical features and consists of over 30 cancer types. Following Kaplan–Meier survival analysis, significant differences between NRP1 low and NRP1 high populations were observed in both LGG and GBM (LGG Log-rank *p* = 0.036, HZ = 0.50; GBM log-rank *p* = 0.040, HZ = 0.57) (Figure 1A, 1B). This result revealed a difference in GB median survival of 3.34 months (Figure 1B). The analysis was also conducted using combined groups to represent glioma of all grades, where significant differences in survival were also observed (Supplementary Figure 1). It should be noted that many of the patients' survival data were omitted in the LGG cohort, which may have skewed results. However, this appears to be consistent between the two populations that are being evaluated.

**Figure 2:**
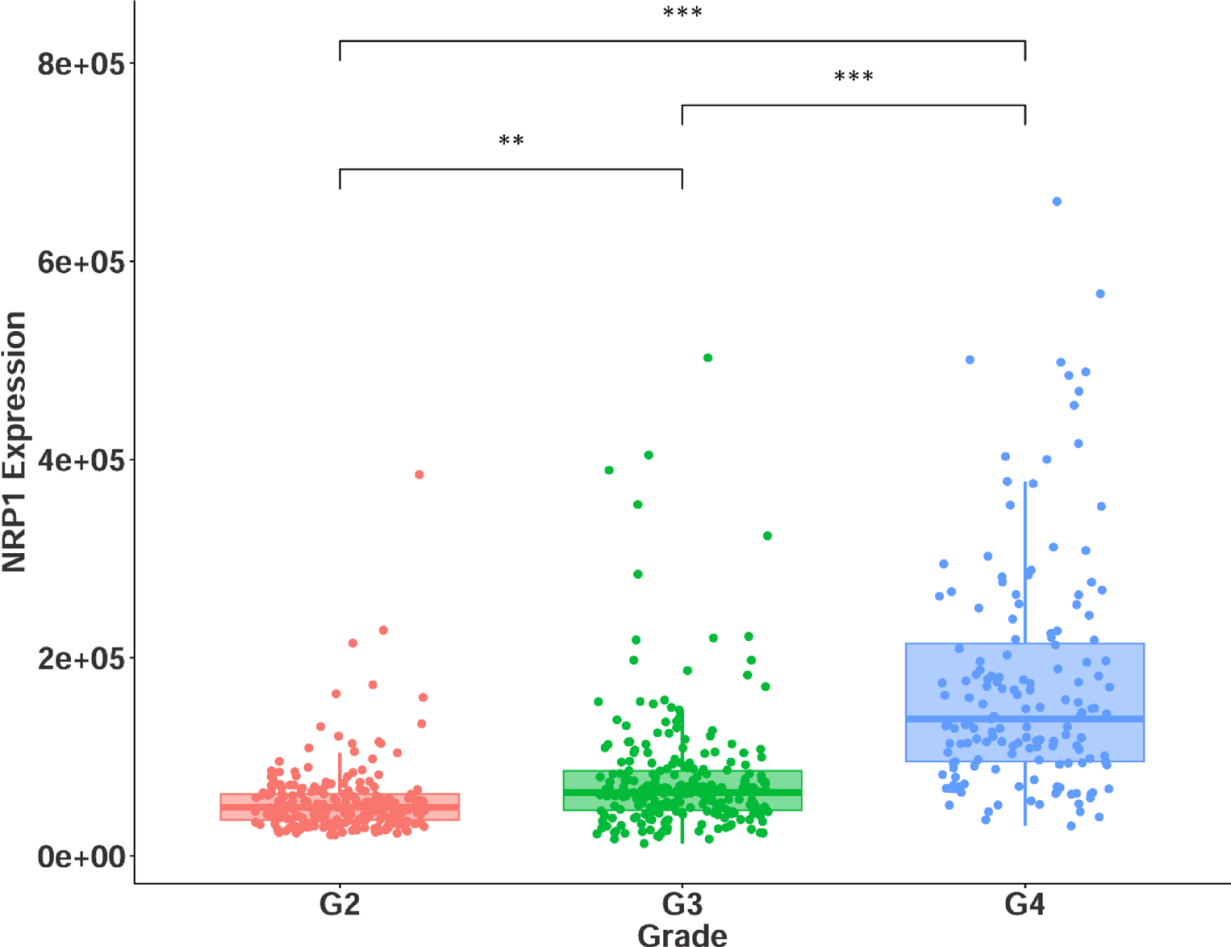
NRP1 expression across glioma grades II-IV G2 = grade II, *n* = 216. G3 = grade III, *n* = 237. G4 = grade IV, *n* = 155. ^**^p < 0.01, ^***^ *p* < 0.001.

Given the differences in survival observed across the two cohorts, and within combined glioma, we next investigated the connection between NRP1 expression and glioma grade. Indeed, NRP1 expression increased significantly as glioma grade increased (Figure 2). This result is in agreement with the conclusions made by Osada *et al.* [22]. Thus, NRP1 expression correlates with poor prognosis and tumor grade in human glioma.

**Figure 3:**
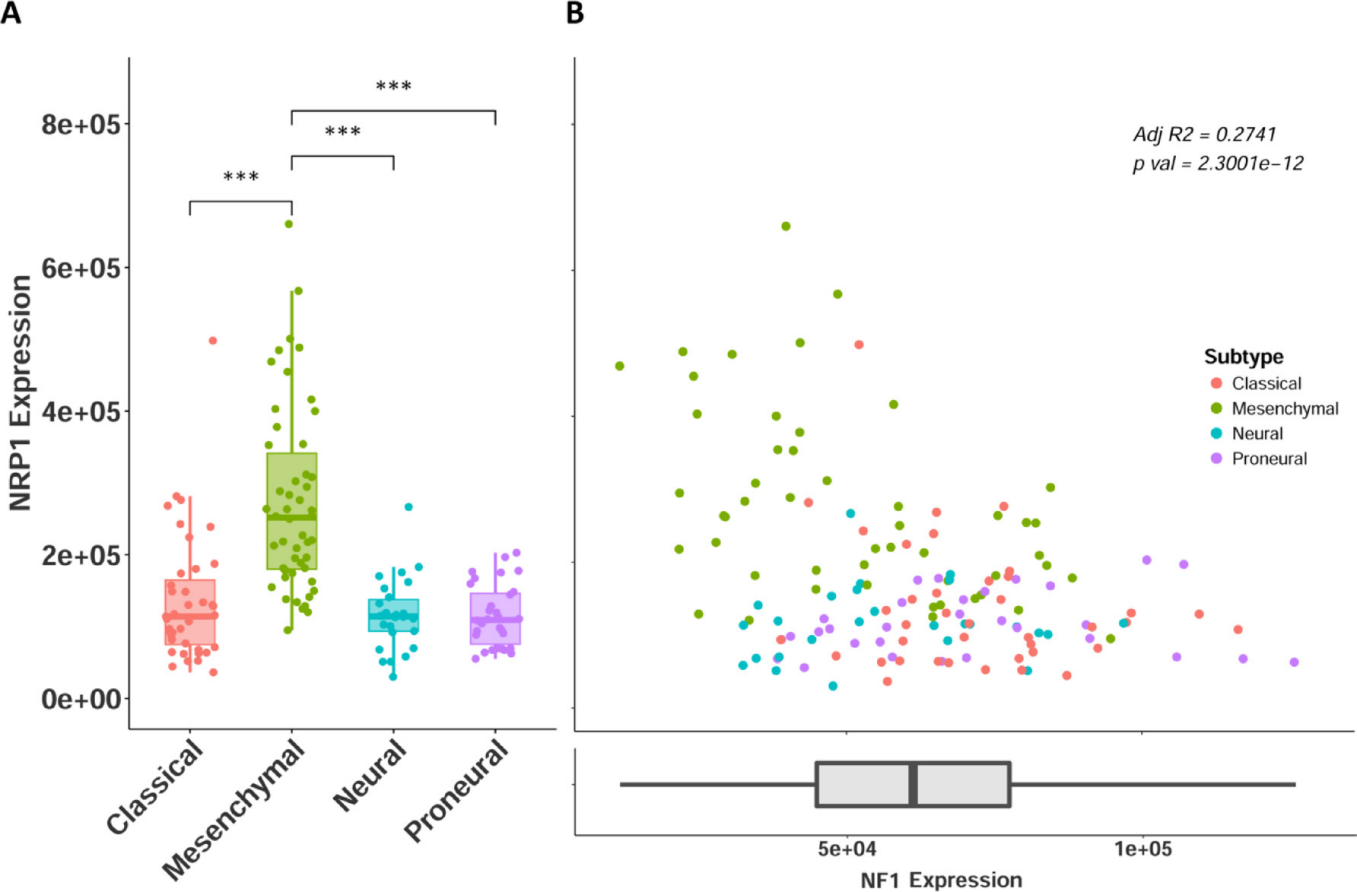
NRP1 expression across GB subtypes (**A**) NRP1 expression separated by molecular subtypes. Classical *n* = 39, Mesenchymal *n* = 50, Neural *n* = 26, Proneural *n* = 30. ^***^p < 0.001. (**B**) NRP1 expression plotted against NF1 expression, colored by subtype. y axis is same as in A. NF1 expression shown as x axis.

Along with histological diagnosis, the WHO now recognizes four distinct molecular subtypes of GB: classical, mesenchymal, neural, and proneural [24–26]. NRP1 expression in the GBM cohort was stratified across the four GB subtypes. NRP1 expression was only found to be significantly upregulated in the mesenchymal group (Figure 3A). The association with the mesenchymal subtype is particularly interesting, as recently, this GB subtype has been determined to be the most aggressive form of GB, has the highest frequency of recurrent transformation, and is linked the recruitment and enrichment of M2 pro-tumorigenic microglia/macrophages [13, 27]. This suggests a potential connection between NRP1, GAMS, and mesenchymal GB. Loss of function of the *NF1* gene distinguishes the mesenchymal subtype, and is functionally related to the recruitment of GAMs in human GB [13, 26]. We therefore sought to examine the correlation between NRP1 and NF1 expression, as this would serve as an additional surrogate for NRP1 association with pro-tumorigenic GAMs. Indeed, there was a significant inverse correlation between NRP1 and NF1 expression in GB, evident in the mesenchymal subtype (Figure 3B). This relationship was not seen in LGG (Supplementary Figure 2), supporting the connection between high NRP1 expression and GB.

**Figure 4:**
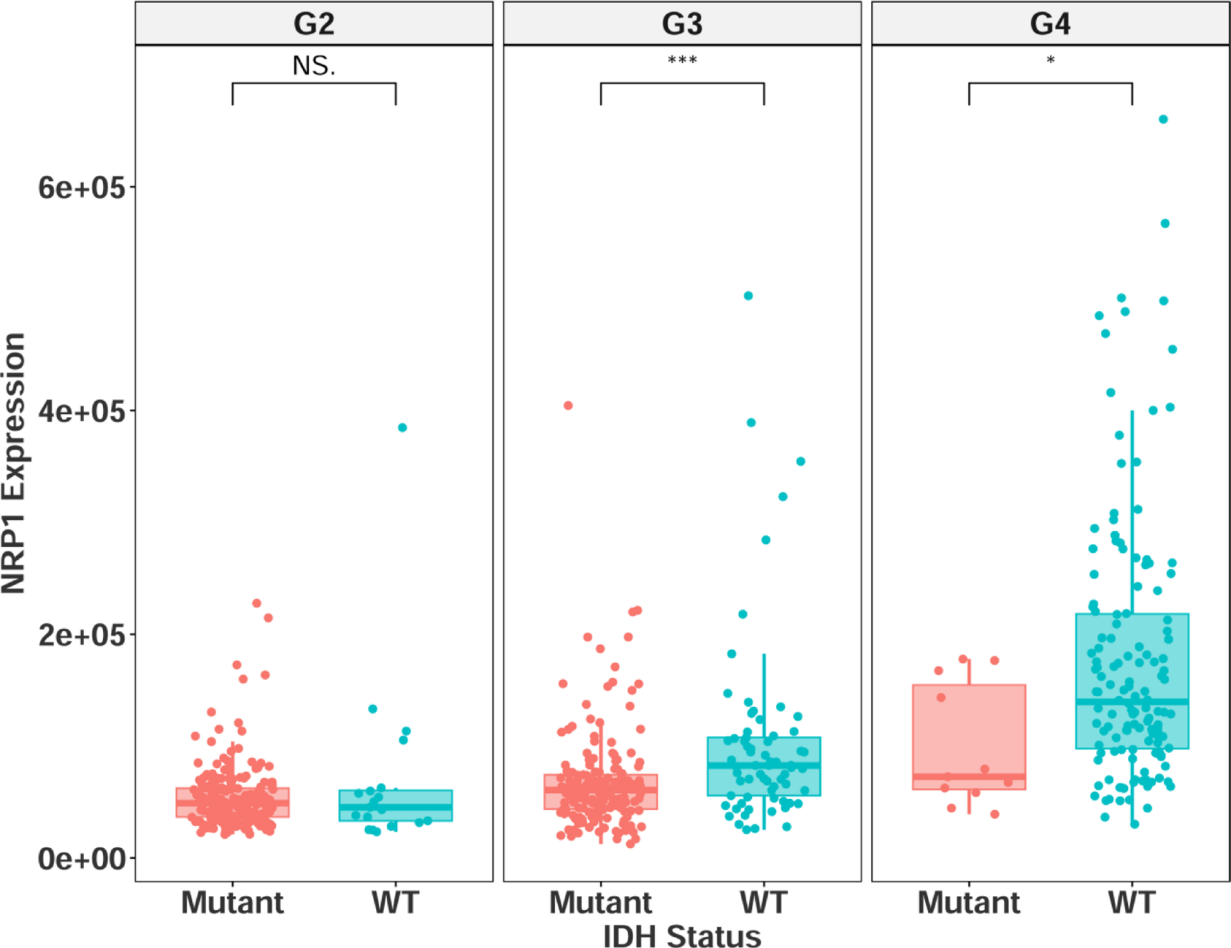
NRP1 expression separated by IDH mutational status in grade II-IV glioma G2 = grade II, mutant = 195, wildtype = 19. G3 = grade III, mutant = 170, wildtype = 67. G4 = grade IV, mutant = 11, wildtype = 140. NS = non-significant, ^*^p < 0.05, ^**^p < 0.01, ^***^p < 0.001.

NRP1 expression was also found to be significantly higher in grade III-IV patients with IDH wild type status (Figure 4), further demonstrating its connection with HGG disease progression. The grade and subtype data were cross-referenced against a recently published database, which included IDH selection criteria, the LGG cohort, and additional, non-TCGA patients (http://recur.bioinfo.cnio.es/) [13], and the query was in agreement with the results reported here, providing further support to an important role of high NRP1 expression. Multivariate analysis was carried out in the NRP1 low and high populations across the combined glioma data set (Supplementary Figure 3). Although NRP1 was not identified as an independent prognostic marker of glioma pathology, our analysis indicates that NRP1 expression in human glioma is inversely correlated with survival as well as clinicopathological features. This suggests a functional role for NRP1. It should be noted that high NRP1 expression was associated with wild-type IDH in grade IV glioma. This may attribute to the decreased hazard ratio following segregation of these variables. Kaplan–Meier analysis of relative NRP1 expression in grade IV IDH wild type patients still suggests a connection between NRP1 and prognosis (Supplementary Figure 4) (GBM IDH WT Log-rank *p* = 0.0886, HZ = 0.62), although, it is not significant in this sample size.

### NRP1 expression is correlated with pro-tumorigenic monocytes/macrophages

**Figure 5:**
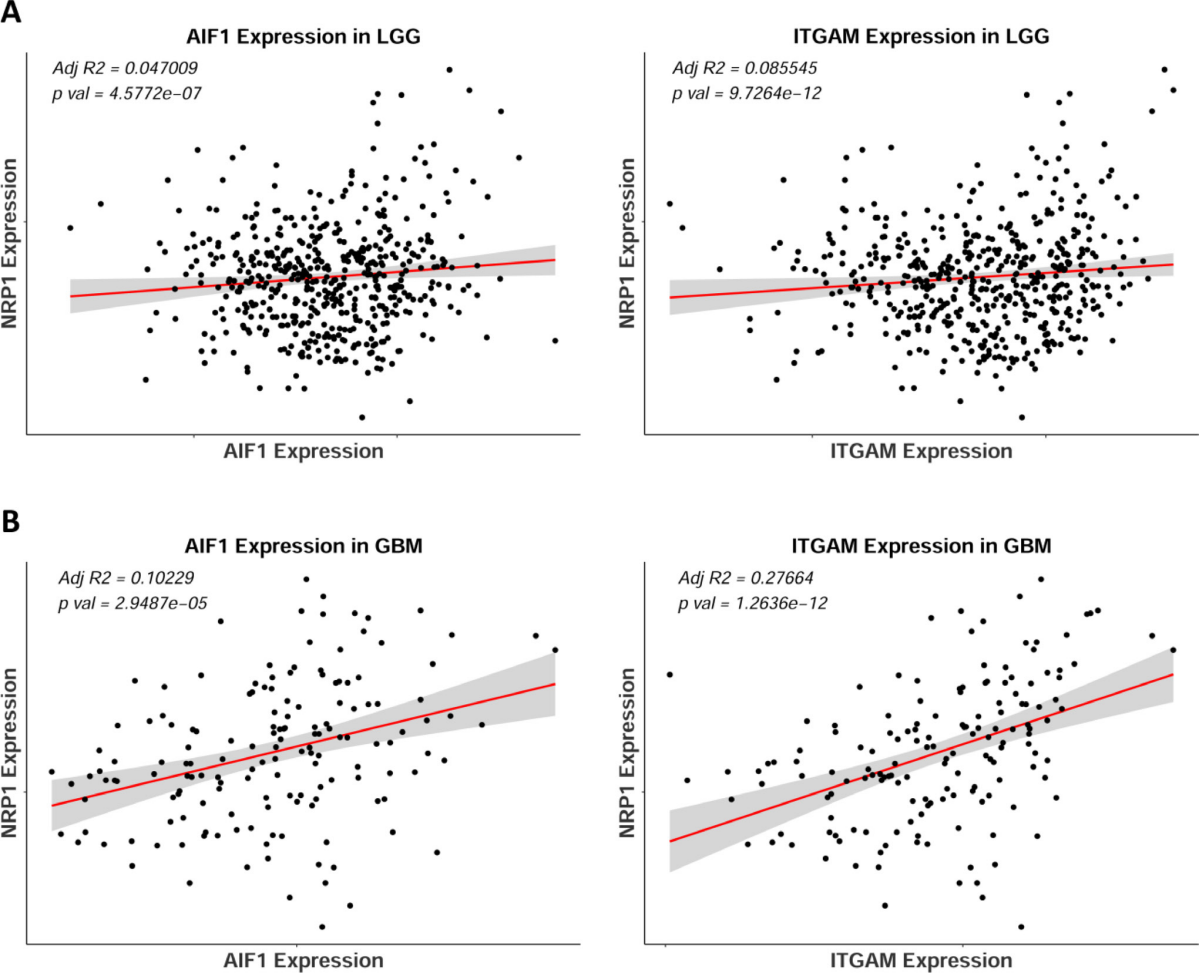
NRP1 expression correlates with monocytic markers AIF1 and ITGAM in human GB (**A**) Linear regression of NRP1 expression against AIF1 and ITGAM expression in LGG, *n* = 510. (**B**) Linear regression of NRP1 expression against AIF1 and ITGAM expression in GBM, *n* = 155.

**Figure 6:**
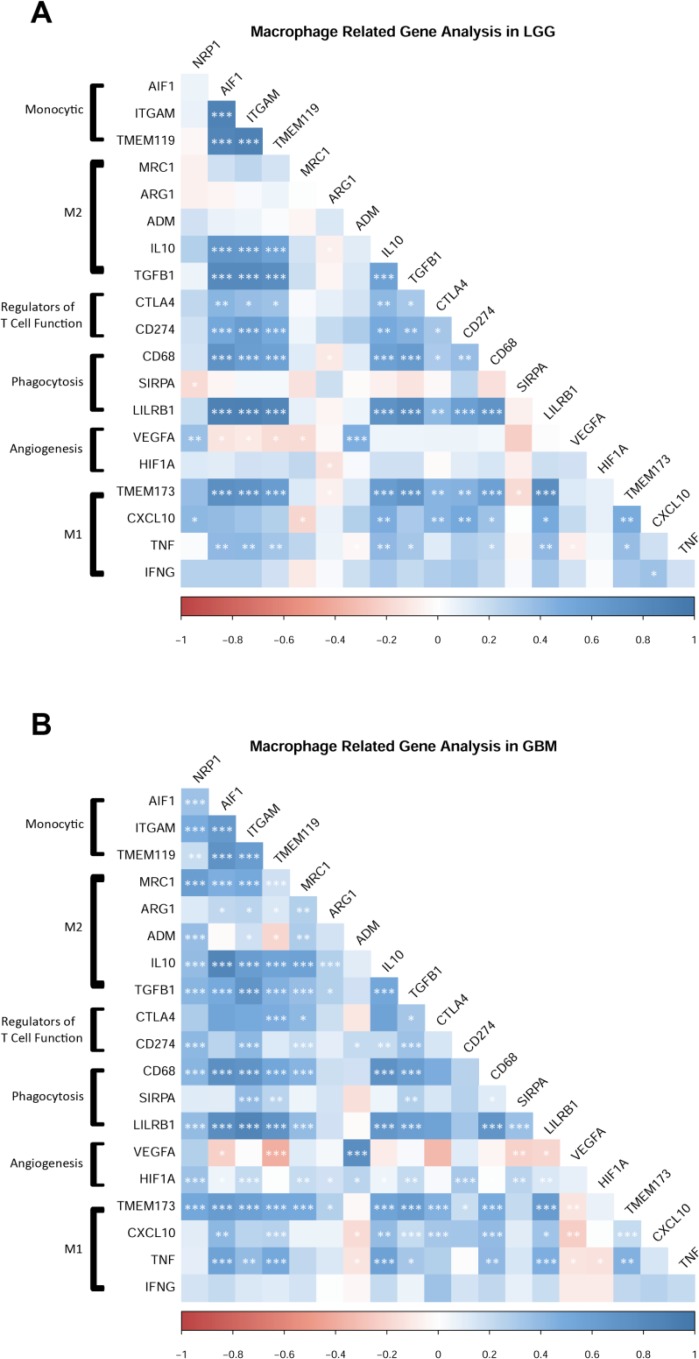
NRP1 expression is associated with the expression of pro-tumorigenic macrophage related genes (**A**) LGG cohort, *n* = 510. (**B**) GBM cohort, *n* = 155. Genes grouped by functional relationships. Correlation values from expression levels are plotted by color. Legend shows correlation values from –1 to 1. Significant correlations between genes are marked. ^*^p < 0.05, ^**^p < 0.01, ^***^p < 0.001.

AIF1 and ITGAM (Iba1 and CD11b, respectively) are pan markers of monocytes, macrophages, and microglia, and are highly upregulated across human GB subtypes [13]. Using these two markers as cellular indicators of possible NRP1 localization, linear regression analysis revealed that both AIF1 and ITGAM were significantly correlated with NRP1 expression in GB patients (Figure 5B). Further examining select genes with distinct functional roles in macrophages, NRP1 expression was found to be significantly correlated with genes that characterize the M2 pro-tumorigenic GAM signature, such as Adm and Mrc1 [16, 17, 28–30], as well as those involved in angiogenic signaling [29, 31], phagocytosis [32, 33], and negative regulation of T cell function [34] in the TCGA GBM cohort (Figure 6B). These relationships were not as robust in LGG (Figures 5A, 6A). Interestingly, there was also a strong correlation with the microglia-specific marker TMEM119 [35]. Microglia are often unable to be defined from infiltrating peripheral macrophages of the TME. Although only a single marker is used here as a proxy, this suggests that microglia could have distinct roles in the TME. There was no significant correlation observed with pro-inflammatory genes CXCL10, TNFα or INFγ. There was however a significant correlation between NRP1 expression and TMEM173. TMEM173 codes for STING protein, which has been associated with both M1 and M2 macrophage phenotypes [36, 37]. Together this functional gene analysis suggests that NRP1 is associated with markers of monocytic infiltration and pro-tumorigenic GAMs in human GB.

**Figure 7:**
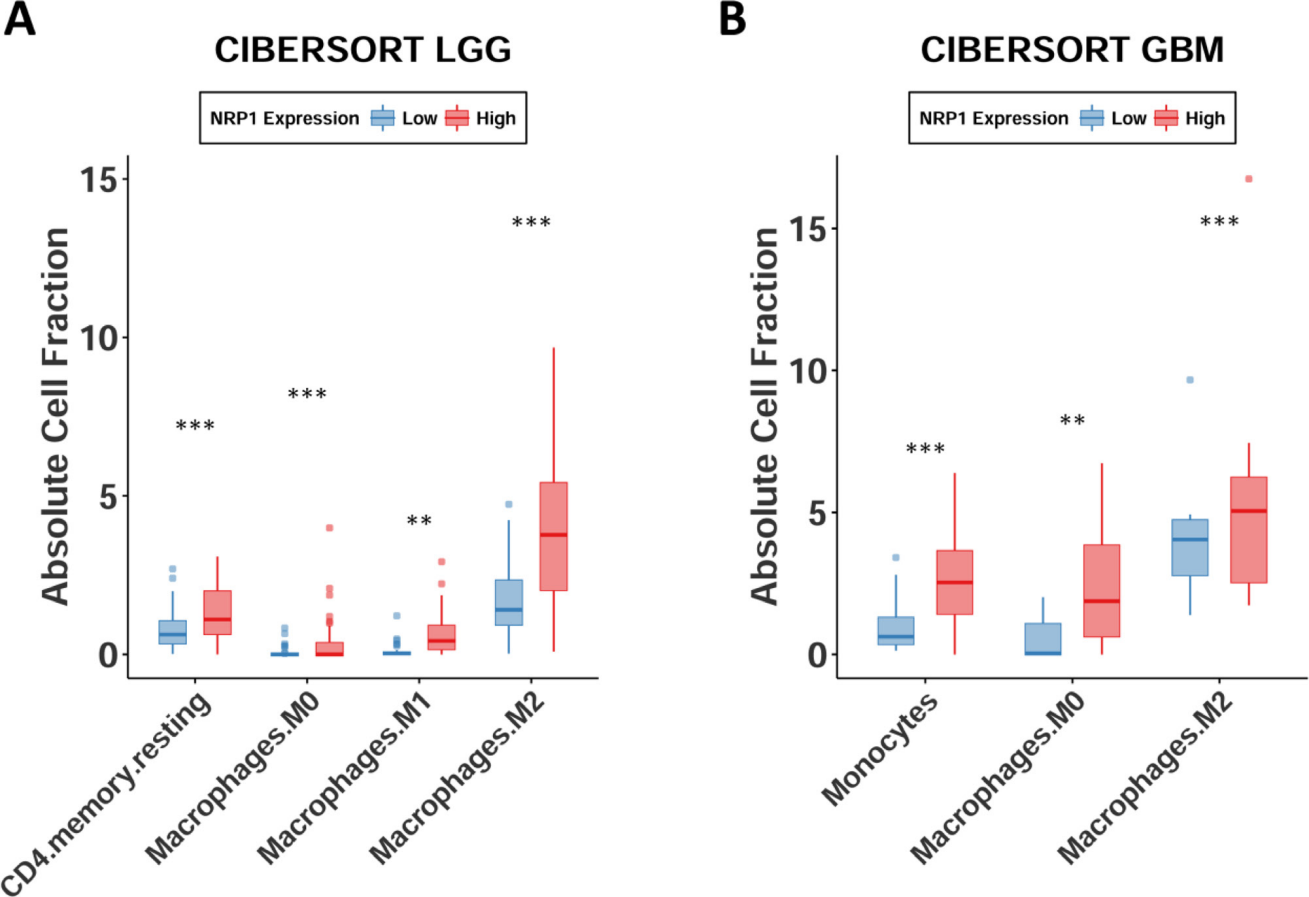
Monocytes, macrophages, and M2 macrophages are enriched in glioma Absolute cell fractions derived from patients' RNA-seq data using CIBERSORT. Patients NRP1 expression segregated by quartiles. Only groups with significant differences are shown. ^*^p < 0.05, ^**^p < 0.01, ^***^p < 0.001.

To further support the hypothesis that NRP1 is primarily expressed by pro-tumorigenic GAMs, and that this may drive disease progression, we used the CIBERSORT *in silico* method to determine absolute immune cell fractions within the TME ((https://cibersort.stanford.edu/) [38]. CIBERSORT uses genetic profiles characteristic for 22 immune cell sub-populations, and can derive relative and absolute cellular fractions in tumor biopsies from transcriptional datasets. Our results demonstrate that both LGG and GB patients with high NRP1 expression have enriched monocytic, macrophage, and M2 macrophage populations (Figure 7A, 7B) (selected comparisons shown for clarity, full analysis can be found in Supplementary Figure 5). It should also be noted that NRP1 is not included in the LM22 gene signature profile list used to distinguish the sub-populations of immune cells, ruling out artificial increases in absolute cell fraction. These results were also confirmed using an additional RNA-Seq deconvolution algorithm, xCell (http://xcell.ucsf.edu/) (data not shown). In conjunction with the above functional gene analysis, there appears to be a strong connection between NRP1 expression and GAMs in human GB. These expression data suggest that the activity of this co-receptor may lead to pro-tumorigenic changes in this innate immune cell sub-population that drive disease progression.

## DISCUSSION

Accompanied by an extremely high mortality rate, GB is associated with histopathological variability and a median survival of approximately 14 months with standard therapy, marking it one of the most lethal cancers [3, 4, 39]. The current treatment modality combines maximal surgical debulking with concomitant temozolomide (TMZ) and radiation therapy. Although this strategy does improve median survival moderately, it has not been updated since its initiation [4].

With the explosive growth of immunotherapies, new tools are arising to treat glioma. Targeting GAMs via CSF1R inhibition is a novel suggested therapy, which has recently completed phase II clinical trials, however, results already exist detailing potential resistance mechanisms [16, 28, 40, 41]. NRP1's role in cancer is increasingly clear, and targeting this co-receptor within the TME, or specifically on GAMs, may prove to be a novel therapeutic strategy. However, the above analyses are only able to *indirectly* support the hypothesis that GAM-specific NRP1 expression plays a major role in GB disease. Follow up studies should be conducted to directly detect the extent that GAM-specific NRP1 correlates with GB survival.

We have shown in the GL261 model of murine GB that GAM-specific knockout of NRP1 slows disease progression by reducing tumor vascularization and inhibiting immunosuppressive TGFβ signaling [20]. M2 pro-tumorigenic monocytes/macrophages are now realized to be prevalent contributors to many solid malignancies. The goal of modulating this cell population often aims to reverse their pro-tumorgenic behavior, and shift the innate immune cells to adopt anti-tumorigenic activity. Although studies manipulating tumor-associated monocytes/macrophages show only small increases in M1-like cytokines and chemokines (such as TNFα, IL1β etc.), the greatest increase was seen following monocyte-specific NRP1 ablation [17, 19, 42]. These studies by others are in agreement with our previous work in murine GB, where NRP1-deficient microglia/macrophages found in the TME displayed lower levels of the M2 marker CD206 (Mrc1) and displayed a higher ratio of cells expressing the classical M1 marker CD86, suggesting a shift towards an M1-like phenotype following inhibition of NRP1 signaling in this cellular population [20]. Hypoxic tumor regions induce potent angiogenic signaling in macrophages [43, 44]. This process regulates the expression of VEGF, contributes to vascular remodeling, and is reportedly dependent on the activity of hypoxia-inducible factor 1-alpha (Hif1a) [19, 29, 42, 45]. We have previously shown that NRP1-deficient bone marrow-derived monocytes that traffic to the TME exhibit a large decrease in expression of Hif1a [21]. This finding is recapitulated in human GB, where NRP1 was found to be highly correlated with Hif1a expression (Figure 6). Thus, NRP1 ablation alone may constitute a ‘two-hit’ mechanism by which these cells slow tumor progression through reversing the M2-like phenotype while simultaneously stunting the neoangiogenic potential of GAMs.

Our present comprehensive analysis of NRP1 expression in human GB, in combination with the conclusions from our murine glioma model [20, 21, 23], suggest that NRP1 is a valid target to pursue in future work. The observed correlation with genes characteristic of pro-tumorigenic GAMs, and the high cellular fractions found in the TME also suggest that there is a strong connection between NRP1 and tumor supporting cell populations. Activity of this co-receptor may lead to pro-tumorigenic changes that significantly contribute to GB disease progression. We therefore propose that NRP1 be considered a prominent target in future studies for GB therapy.

## MATERIALS AND METHODS

Patient and RNA-seq data were obtained through the NCI's Genomic Data Commons repository (https://portal.gdc.cancer.gov/) by accessing TCGA datasets from the LGG and GBM studies [46, 47]. Of the 529 RNA-Seq samples listed in the LGG study, 511 cases were selected based on primary solid tumor samples. Of the 174 RNA-Seq samples listed in the GBM study, 156 cases were selected based on primary solid tumor samples. Patients without complete clinical data sets were then excluded. All statistical analysis and graphing were conducted in R for Windows (https://www.r-project.org/). For relative expression comparisons, NRP1 expression levels were distinguished into upper and lower quartiles. This resulted in 128 patients per group from the LGG cohort, and 39 patients per group from the GBM cohort.

The Kaplan–Meier method and Cox regression analysis were used for survival comparisons. This statistically compares overall survival between groups and generates a hazard ratio which describes the likelihood of death occurring. For grade, subtype, IDH status, and gene comparisons, expression was taken across the entire patient sample set, as a less biased approach then segregating into quartiles. One-way ANOVA with Tukey's post hoc analysis was used for grade, subtype, and IDH comparisons. AIF1 and ITGAM expression were fitted with a linear regression model and compared for statistical significance. The R package *corrplot* was used to generate correlation values between the expression of 19 genes selected based on functional relevance. CIBERSORT analysis was performed using the online software (https://cibersort.stanford.edu/). This algorithm compares gene expression data sets to that of 22 immune cell subtype gene signature profiles to derive relative and absolute fractions of cell populations within the tumor sample. Two-way ANOVA with Bonferroni's correction was used for CIBERSORT comparisons.

## SUPPLEMENTARY MATERIALS FIGURES


