# Supplementary material for: Expression of neuropilin-1 is linked to glioma associated microglia and macrophages and correlates with unfavorable prognosis in high grade gliomas
Source: Oncotarget. 2018 Nov 2;9(86):35655–65. doi: 10.18632/oncotarget.26273 (PMC6235016; doi:10.18632/oncotarget.26273)
Supplement: Supplementary file 1 [file oncotarget-09-35655-s001.pdf]

Expression of neuropilin-1 is linked to glioma associated microglia and macrophages and correlates with unfavorable prognosis in high grade gliomas

SUPPLEMENTARY MATERIALS

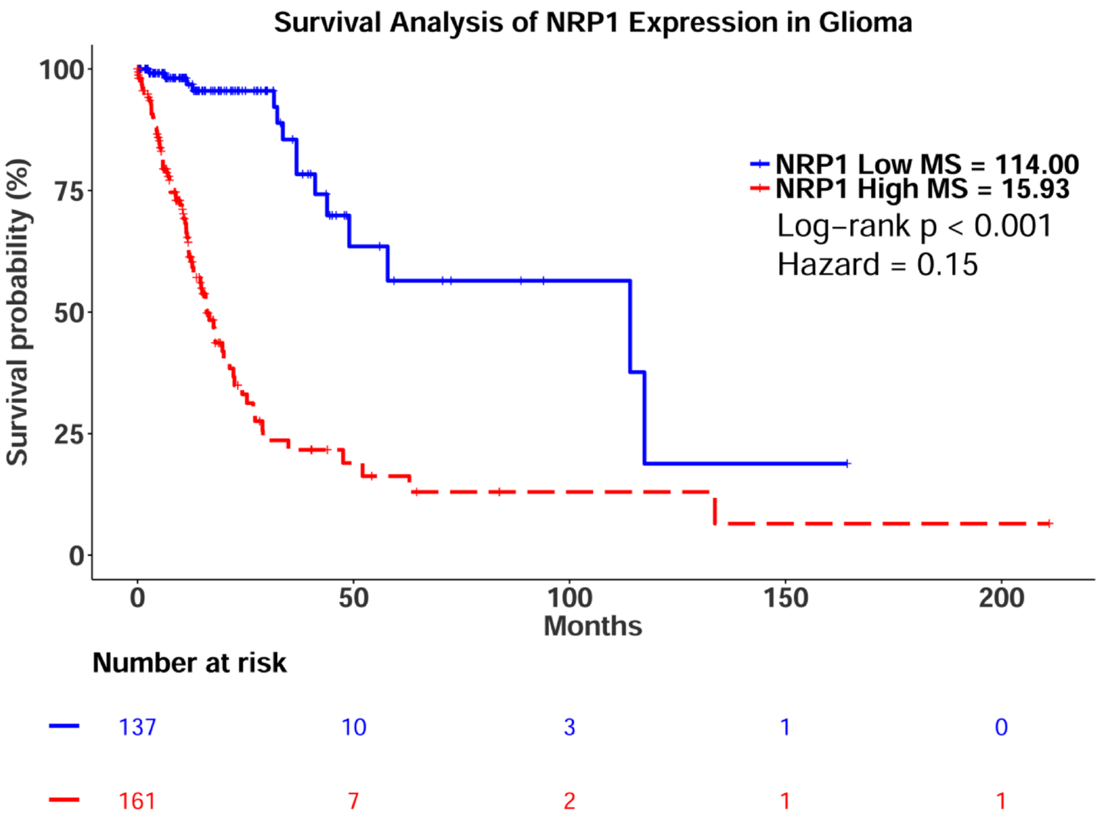

Supplementary Figure 1: Kaplan–Meier survival analysis of relative NRP1 expression in combined LGG and GBM groups. NRP1 Low median survival = 114 months, NRP1 High median survival = 15.93 months.

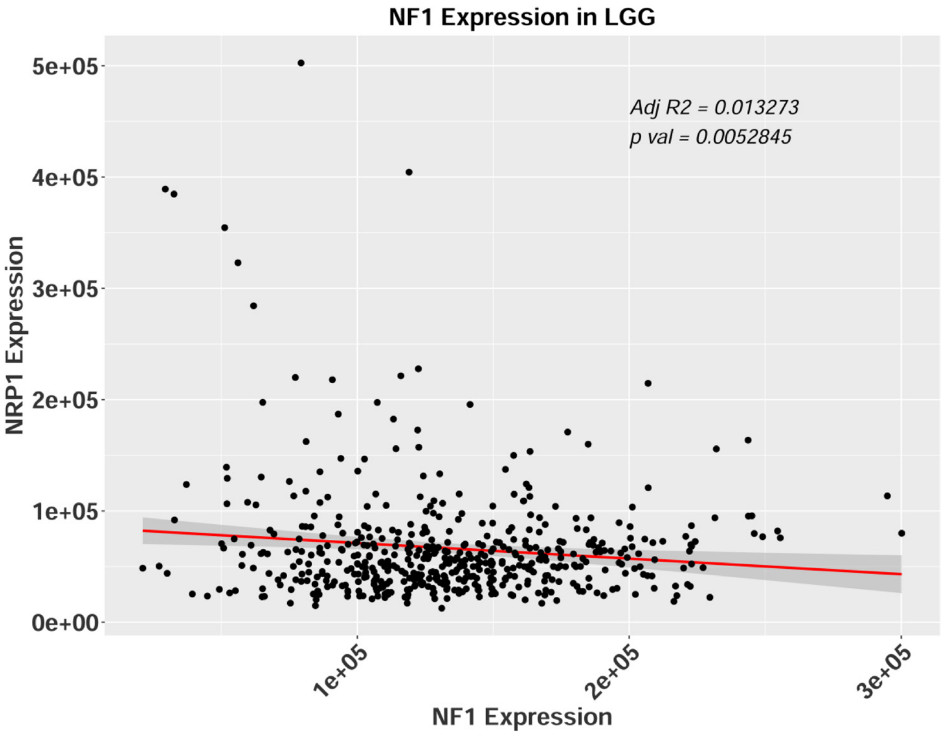

Supplementary Figure 2: Linear regression of NRP1 expression against NF1 expression in LGG.

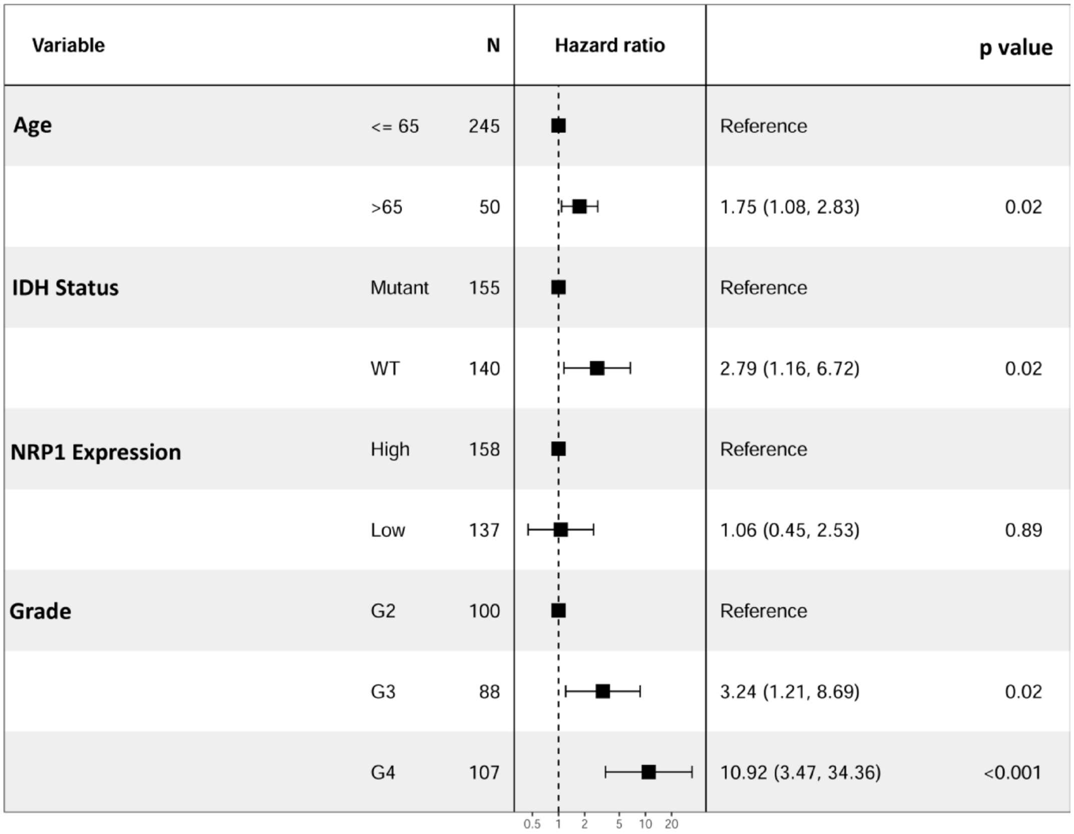

Supplementary Figure 3: Forrest plot following multivariate analysis of prognostic markers in glioma. Corrected hazard ratios and *p* values shown.

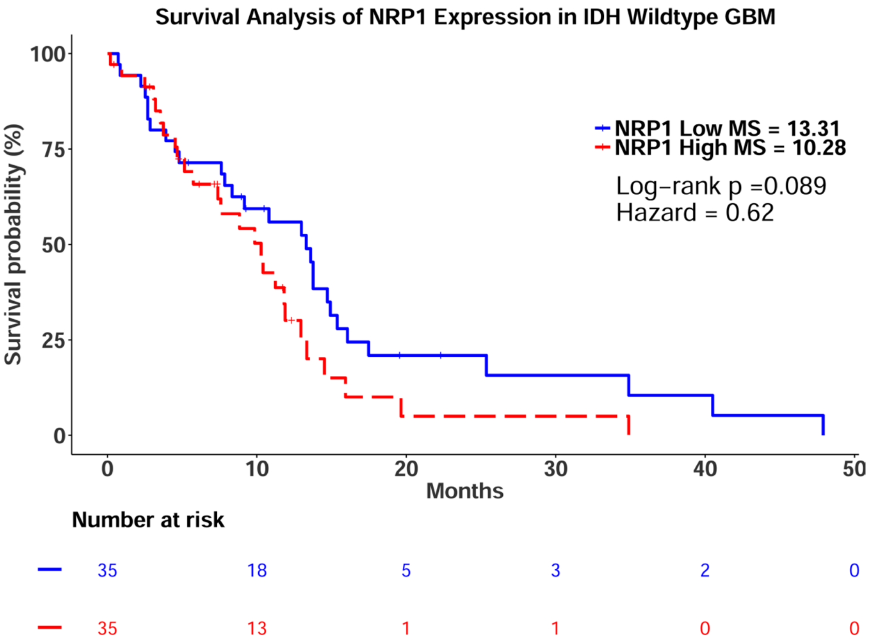

Supplementary Figure 4: Kaplan–Meier survival analysis of relative NRP1 expression in GBM in IDH wild type patients. NRP1 Low median survival = 13.31 months, NRP1 High median survival = 10.28 months.

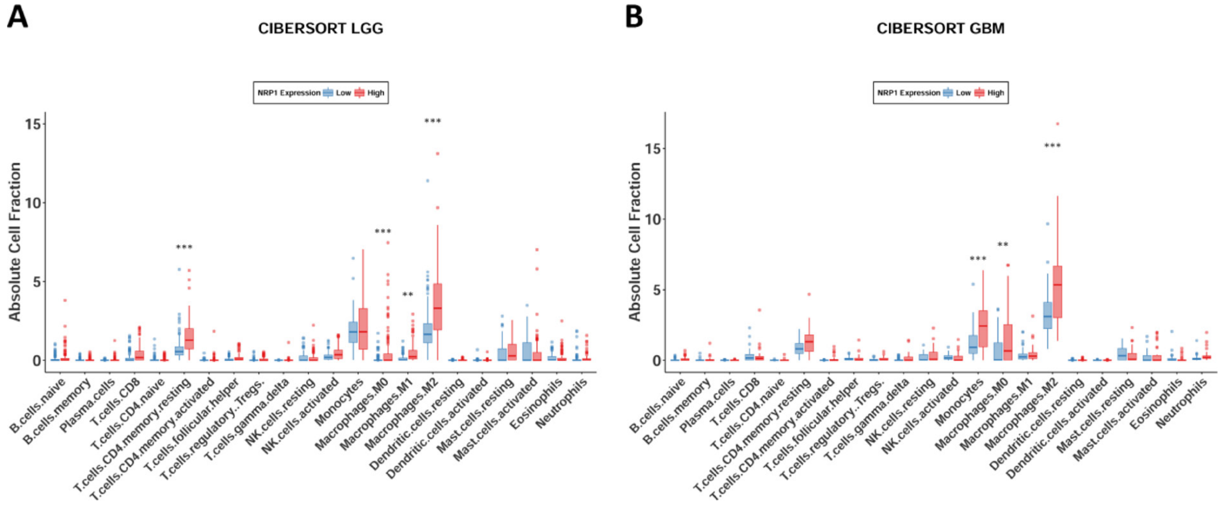

Supplementary Figure 5: CIBERSORT results of relative NRP1 expression in LGG and GB. Absolute cell fractions of 22 immune cell populations derived from patients' RNA-Seq data. $^{*}p < 0.05$, $^{**}p < 0.01$, $^{***}p < 0.001$.
